# Supplementary material for: Single-cell transcriptome analysis reveals aberrant stromal cells and heterogeneous endothelial cells in alcohol-induced osteonecrosis of the femoral head
Source: Commun Biol. 2022 Apr 6;5:324. doi: 10.1038/s42003-022-03271-6 (PMC8987047; doi:10.1038/s42003-022-03271-6)
Supplement: Supplementary file 2 — Supplementary Information (new) [file 42003_2022_3271_MOESM2_ESM.pdf]

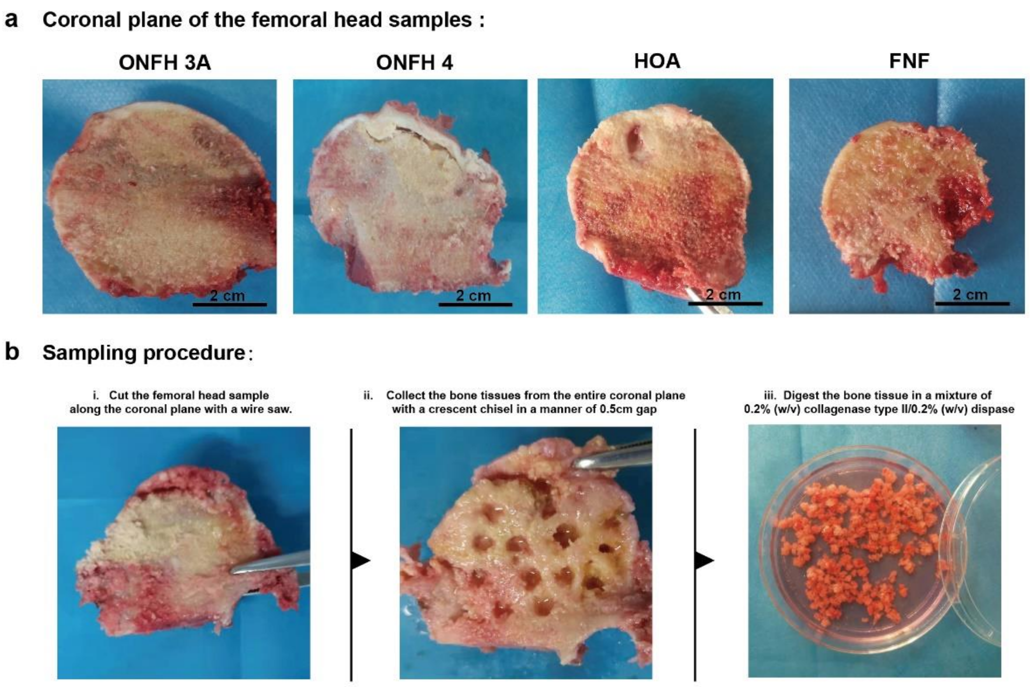

Supplementary Figure 1: Sampling procedure of bone tissue acquisition from femoral head samples. a) Coronal plane of the femoral head samples from different groups. **b)** Specific sampling procedure of bone tissue acquisition. i. Cut the femoral head sample along the coronal plane with a wire saw. ii. Collect the bone tissues from the entire coronal plane with a crescent chisel in 0.5 cm gap. iii. Digest the bone tissue in a mixture of 0.2% (w/v) collagenase type II / 0.2% (w/v) dispase.

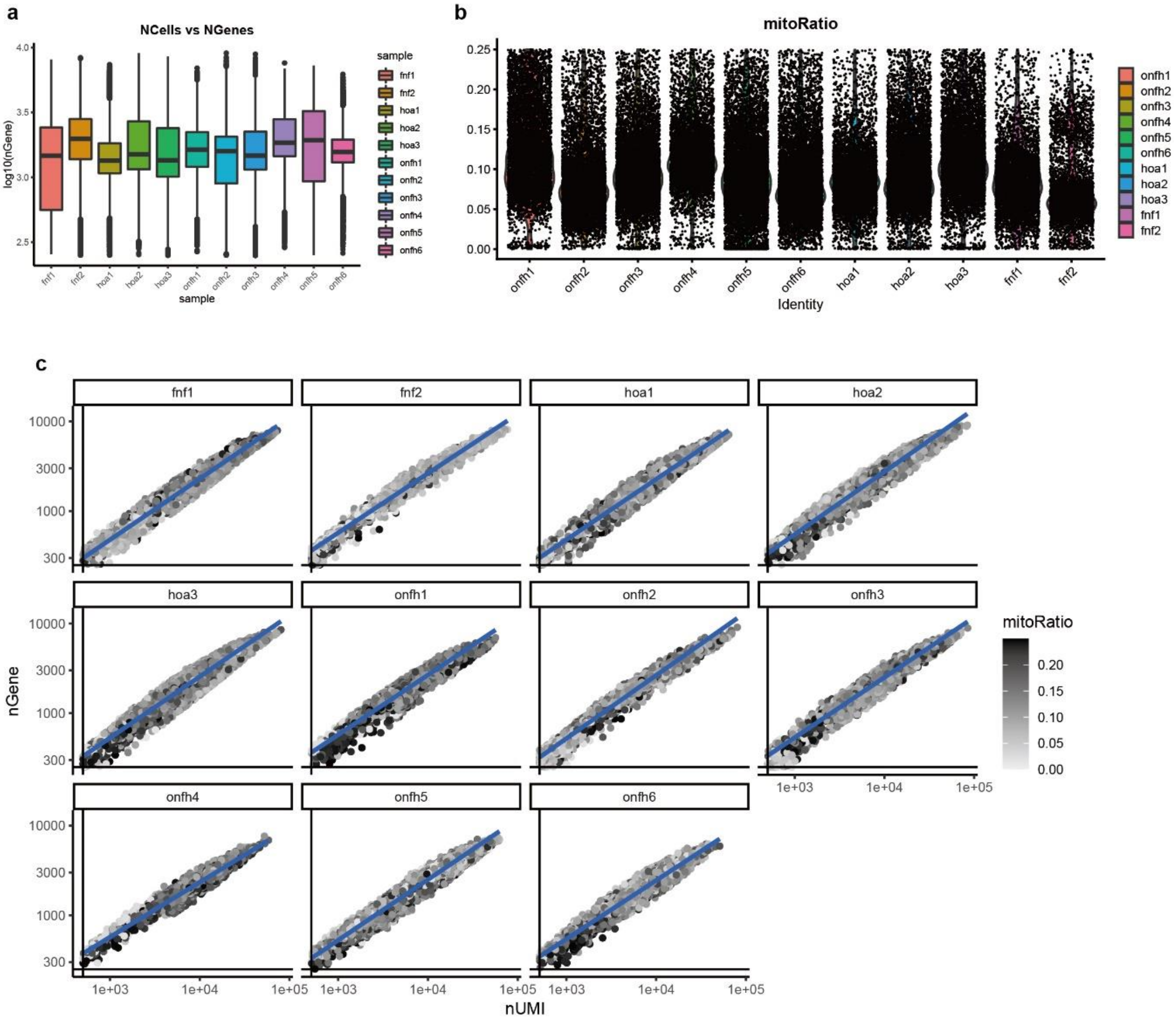

Supplementary Figure2: Quality control of sequencing data. a) Box plot shows the average number of genes detected in a single cell of each sample. **b)** Violin pot shows the ratio of mitochondrial related genes to all detected genes in each cell. Each dot represents a cell. **c)** Cowplot shows the correlation between the number of genes detected per cell and the number of UMI (unique molecular identifiers). Each dot represents a cell. The gray depth reflects the proportion of mitochondrial related genes.

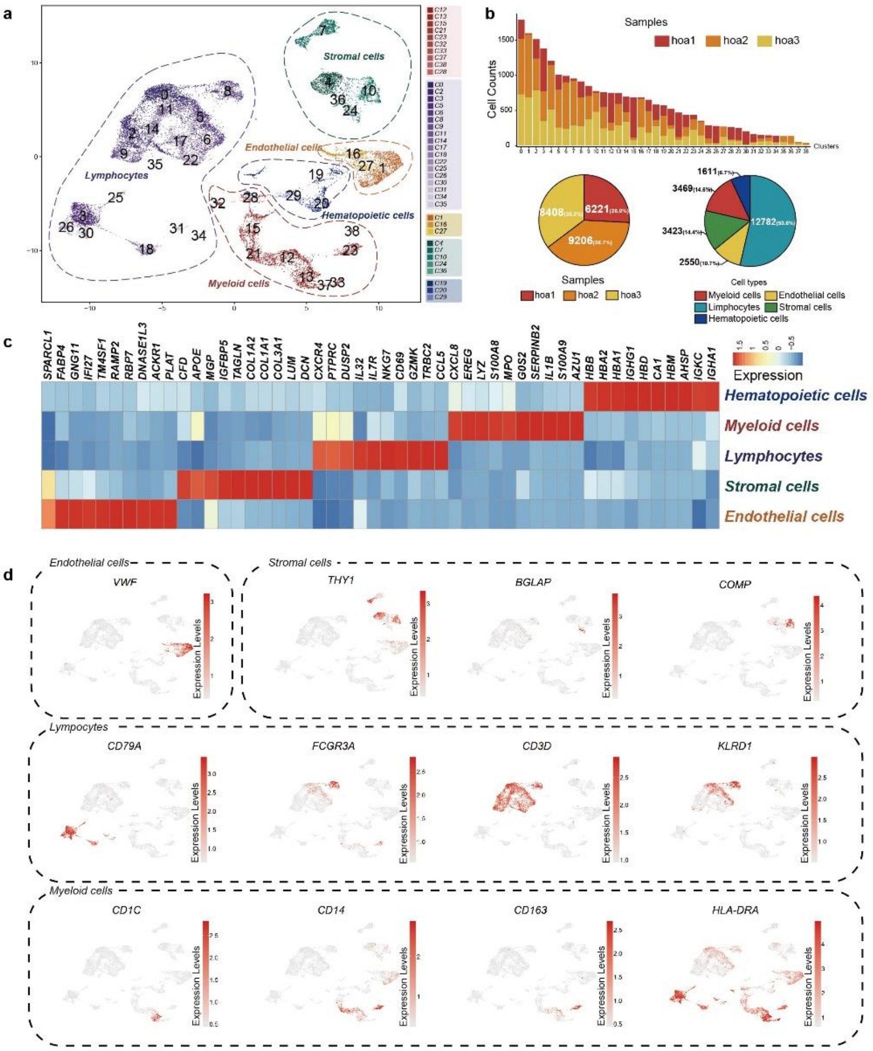

Supplementary Figure 3: Landscape of the regional cells on HOA femoral heads. a) UMAP plot based on 23,835 high quality regional cells on HOA femoral heads. The cells were grouped into 35 clusters, consisting of 5 major groups of cells (MCs: myeloid cells, LCs: lymphocytes, ECs: endothelial cells, SCs: stromal cells and HCs: Hematopoietic cells). **b)** Bar plot and pie charts showing the cell composition within each cell cluster, HOA samples and 5 major clusters. **c)** Top 10 DEGs of the 5 major cell subsets. **d)** UMAP plots representing the expression value of recognized markers of the 5 major cell subsets in HOA cells.

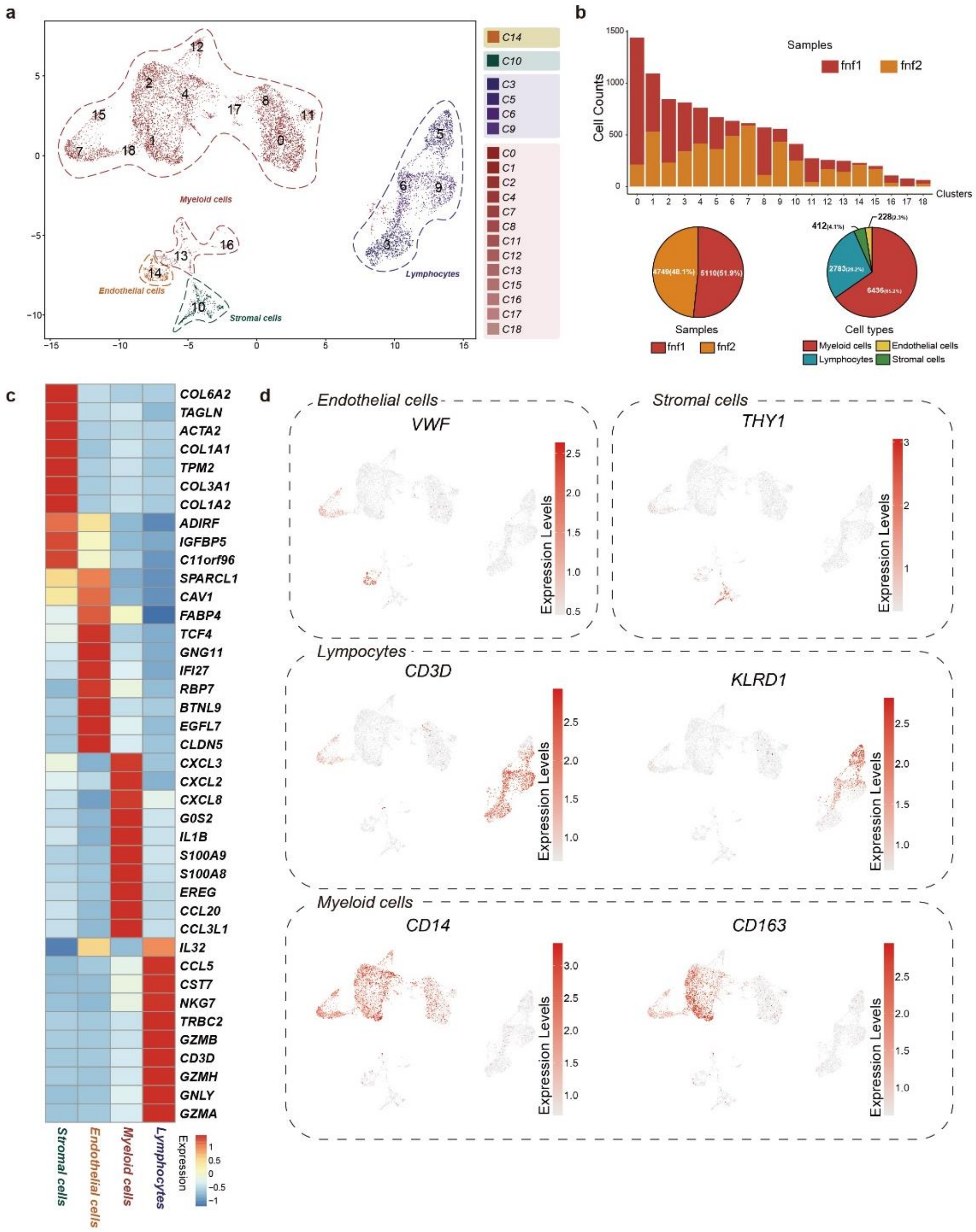

Supplementary Figure 4: Landscape of the regional cells on FNF femoral heads. a) UMAP plot based on 9,859 high quality Regional cells on FNF femoral heads. The cells were grouped into 19 clusters, consisting of 4 major groups of cells (MCs: myeloid cells, LCs: lymphocytes, ECs: endothelial cells and SCs: stromal cells). **b)** Bar plot and pie charts showing the cell composition within each cell cluster, FNF samples and 4 major clusters. **c)** Top 10 DEGs of the 4 major cell subsets. **d)** UMAP plot representing the expression value of recognized markers of the 4 major cell subsets in FNF cells.

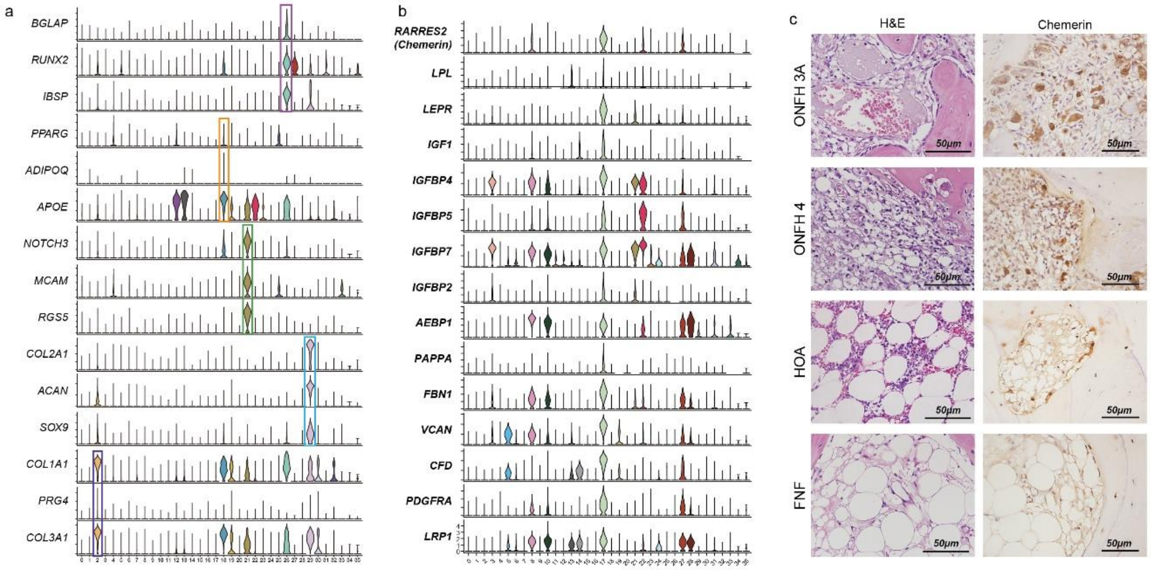

Supplementary Figure 5: Identification of vacuolar cells in bone marrow of the ONFH group. a) Violin plot showing the expression of adipocyte related genes in each cluster of the ONFH group. **b)** Representative bone marrow H&E staining images (left column) and chemerin IHC staining images (right column) for each group, scale bar = 50 μm.

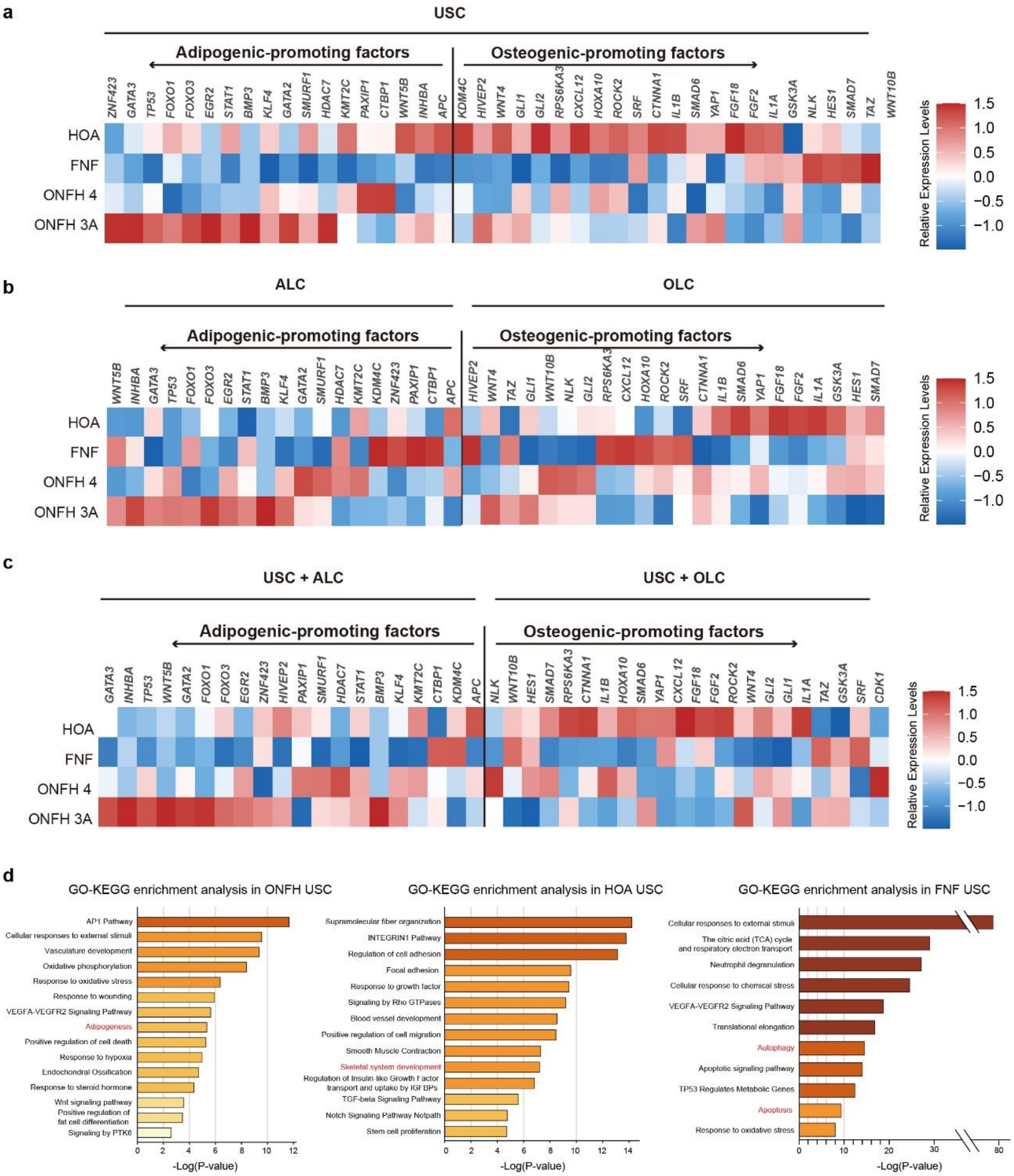

Supplementary Figure 6: Diversity in osteogenic-adipogenic differentiation of SCs between different groups. a-c) Heatmap showing the relative expression levels of recognized osteogenic-promoting or adipogenic-promoting factors in the collection composed of USCs, ALCs and OLCs. **d)** Bar plot showing the GO and KEGG enrichment results for the DEGs ($P < 0.05$, log2-fold change > 0.25) of the MSCs. Enrichment analysis was performed using Metascape. The ONFH group includes stage 4A and 4.

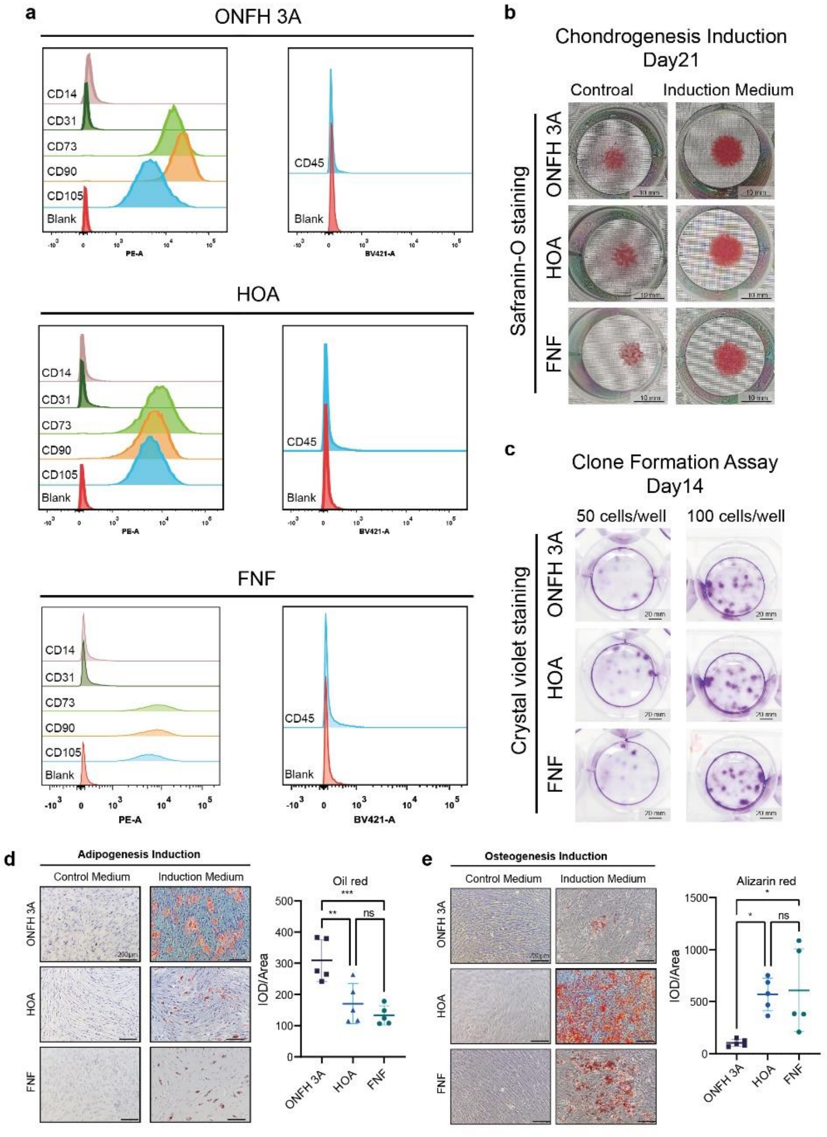

Supplementary Figure 7: Identification and Comparison of Differentiation Competency in MSCs from different groups. a) Flow cytometry assays of classic markers of MSC. **b)** Representative red O staining images of each group of MSC at day 14 of chondroblast differentiation. Scale bar = 10 mm **c)** Representative crystal violet staining images of each group of MSC at day 14 of clone formation experiment. Cells in each group were seeded in 6-well culture plates at 50/well and 100/well, respectively. Scale bar = 20 mm. **d)** Representative oil red O-stained images and quantitative analysis of different groups of MSCs on day 14 of adipogenic differentiation. Scale bar = 200 µm. Data are represented as mean ± SD, n = 5, One-way ANOVA with Tukey's test, ** P<0.01, *** P<0.001. **e)** Representative alizarin red-stained images and quantitative analysis of different groups of MSCs on day 14 of osteogenic differentiation. Scale bar = 200 µm. Data are represented as mean ± SD, n = 5, one-way ANOVA with Tukey's test, * P<0.05.

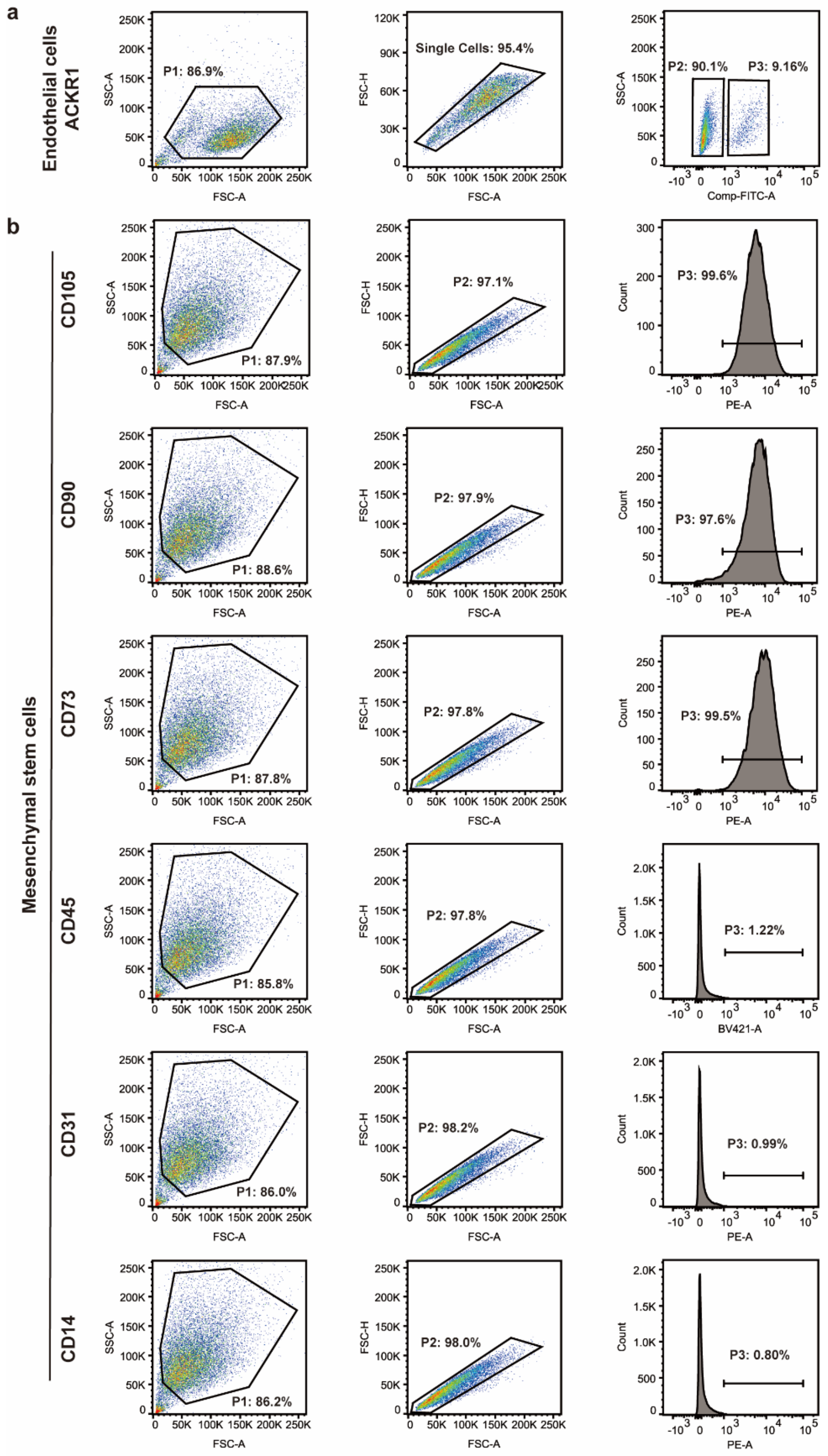

Supplementary Figure 8: Gating Strategy of the flow cytometry for ACKR1+/- ECs and mesenchymal stem cells. a) Flow cytometry gating strategy for ACKR1+/- ECs. P1: nucleated cells; P2: ACKR1- ECs; P3: ACKR1+ ECs. **b)** Flow cytometry gating strategy for mesenchymal stem cells. P1: nucleated cells; P2: single cells; P3: positive cells.

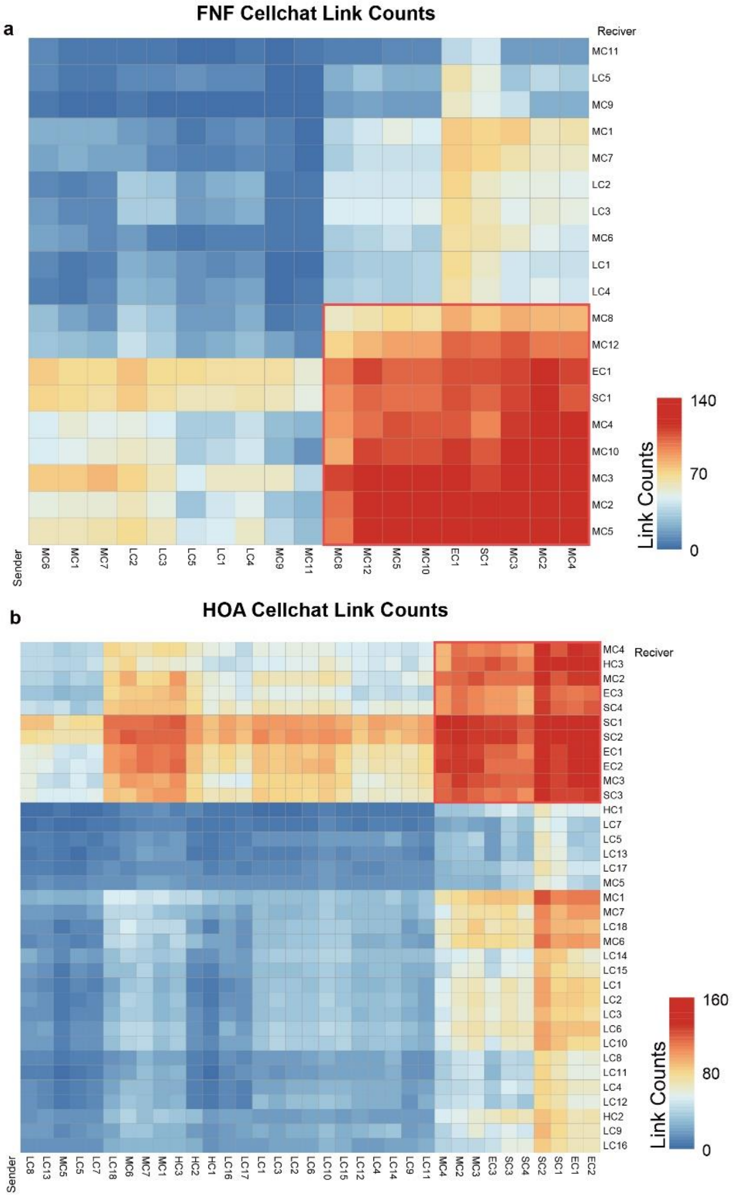

Supplementary Figure 9: Analysis of communication ligand-receptor counts in the HOA and FNF groups. a-b) Heatmap showing the total counts of significant ligand-receptor pairs across all clusters in the HOA and FNF groups. The red frame and blue frame indicate that EC, SC and myeloid cells harbored high-rank numbers of ligand-receptor pairs.

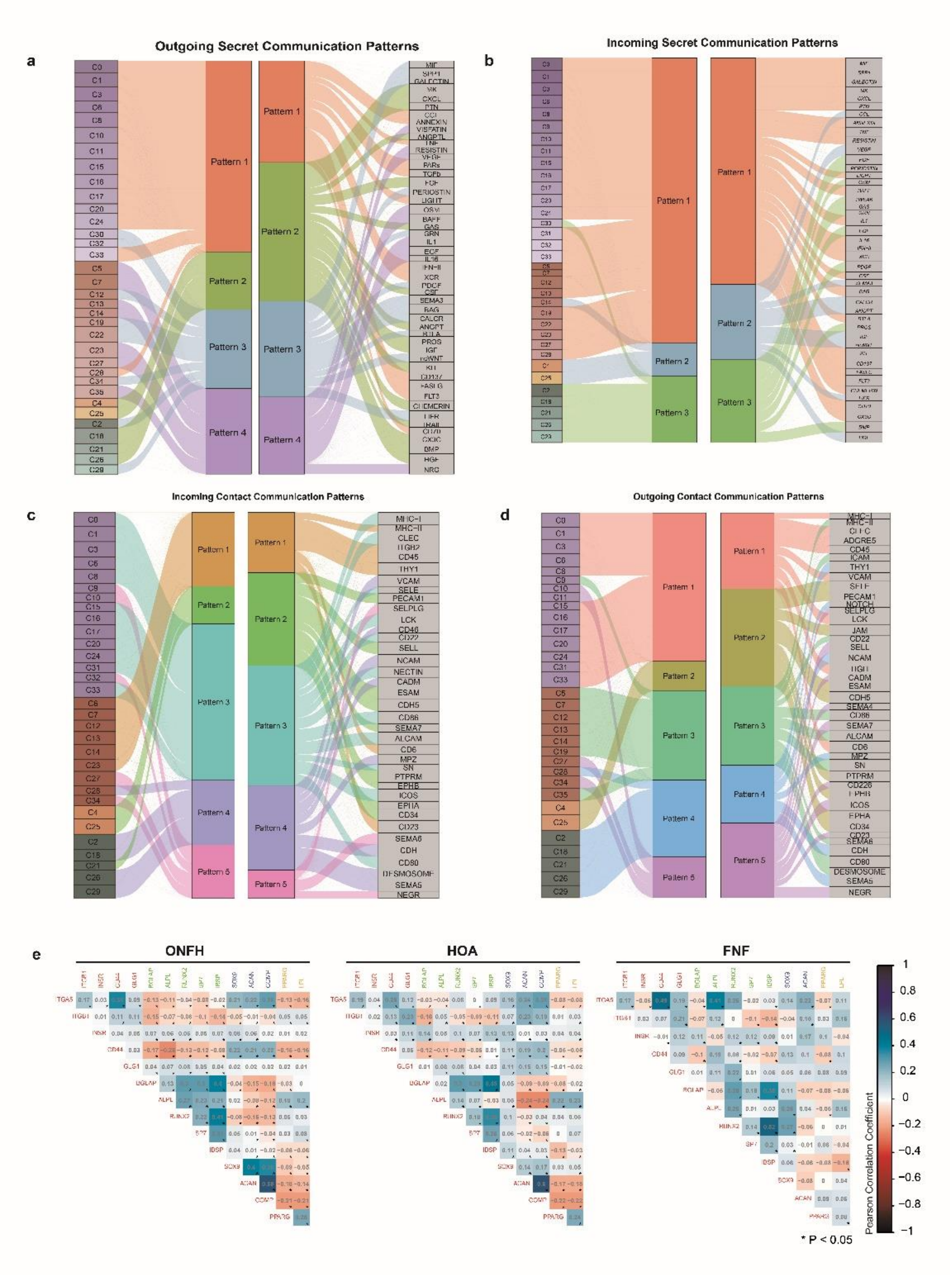

Supplementary Figure 10: Communication pathway analysis and correlation analysis in ONFH. a-d) River plots indicating incoming communication patterns of all clusters. The left column represents the incoming signaling pathways influencing cell groups, the middle column represents the associations of latent patterns with cell groups and signaling pathways, and the right column represents the clusters receiving pathway influence. **e)** Pearson correlation analysis between pathway receptors of interest and adipogenesis or osteogenesis genes in SCs among the three groups. Pearson-test, * P<0.05

Supplementary Table 1. Details of the sample donors

Sample ID	Diagnosis	Stage	Age (years)	Sex
ONFH1[a]	Alcohol-induced ONFH	ARCO[b] 3A	57	Male
ONFH2	Alcohol-induced ONFH	ARCO 3A	36	Male
ONFH3	Alcohol-induced ONFH	ARCO 3A	46	Male
ONFH4	Alcohol-induced ONFH	ARCO 4	54	Male
ONFH5	Alcohol-induced ONFH	ARCO 4	43	Male
ONFH6	Alcohol-induced ONFH	ARCO 4	43	Male
HOA1[c]	Osteoarthritis of the hip secondary to hip dysplasia	KL[d] 3	64	Female
HOA2	Osteoarthritis of the hip secondary to hip dysplasia	KL 4	56	Female
HOA3	Osteoarthritis of the hip secondary to hip dysplasia	KL 4	54	Female
FNF1[e]	FNF	Garden 4	67	Male
FNF2	FNF	Garden 4	62	Male

a Osteonecrosis of the femoral head
b Association Research Circulation Osseous
c Hip osteoarthritis
d Kellgren-Lawrence
e Femoral neck fracture

Supplementary Table 2. Software Information

Name	Identifier	Source
Cell Ranger 3.0	10x Genomics	https://10xgenomics.com/
Seurat 3.2.0	PMID 29608179	https://satijalab.org/seurat/
Monocle 2.16.0	PMID 24658644	https://bioconductor.org/packages/monocle/
SingleR 1.2.4	PMID 30643263	https://bioconductor.org/packages/SingleR/
scCATCH 2.1	PMID 32062421	https://github.com/ZJUFanLab/scCATCH
psych 2.0.7	William Revelle	https://CRAN.R-project.org/package=psych
ClusterProfiler 3.18.1	PMID: 22455463	https://bioconductor.org/packages/clusterProfiler
Metascape 2021.07	PMID: 30944313	http://metascape.org/
GENIE3 1.10.0	PMID: 20927193	https://bioconductor.riken.jp/packages/GENIE3
RcisTarget 1.8.0	DOI: 10.18129/B9.bioc.RcisTarget	http://bioconductor.org/packages/RcisTarget/
AUCell 1.10.0	DOI: 10.18129/B9.bioc.AUCell	https://bioconductor.org/packages/AUCell/
SCENIC 1.2.4	PMID: 28991892	https://github.com/aertslab/SCENIC
CellChat 0.0.2	Https://doi.org/10.1101/2020.07.21.214387	https://github.com/sqjin/CellChat
SoupX 3.2.0	PMID: 33367645	https://github.com/constantAmateur/SoupX
ImageJ 1.52v	NIH	https://imagej.nih.gov/ij/

Supplementary Table 3. Antibodies information

Reagent name	Source	Identifier	Dilution/ Concentration
Rabbit monoclonal anti-ACKR1 (EPR5205)	Abcam	ab137044	1: 200/ 3.25μg/ml
Mouse monoclonal anti-vWF (F8/86)	Invitrogen	MA5-14029	1:100/ 2μg/ml
Rabbit polyclonal anti-Chemerin	Invitrogen	PA5-77080	1:100/ 1μg/ml
PE anti-human CD105 (SN6h)	Biolegend	800503	1:100
PE anti-human CD90 (5E10)	Biolegend	328109	1:100
PE anti-human CD73 (AD2)	Biolegend	344003	1:100
Brilliant Violet 421™ anti-human CD45 (HI30)	Biolegend	304031	1:100
PE anti-human CD31 (WM59)	Biolegend	303105	1:100
PE anti-human CD14 (63D3)	Biolegend	367103	1:100
Goat Anti-rabbit IgG H&L/Cy3 antibody	Bioss	bs-0295G-Cy3	1:500/ 2μg/ml
Goat Anti-Mouse IgG H&L/FITC antibody	Bioss	bs-0296G-FITC	1:500 2μg/ml
