# Supplementary material for: Single-cell transcriptome analysis reveals aberrant stromal cells and heterogeneous endothelial cells in alcohol-induced osteonecrosis of the femoral head
Source: Commun Biol. 2022 Apr 6;5:324. doi: 10.1038/s42003-022-03271-6 (PMC8987047; doi:10.1038/s42003-022-03271-6)
Supplement: Supplementary file 3 — Description of Additional Supplementary Files [file 42003_2022_3271_MOESM3_ESM.pdf]

Description of Additional Supplementary Files

File name: Supplementary Data 1
Description: The top 20 DEGs in alcohol-induced ONFH.

File name: Supplementary Data 2
Description: Top 100 GO terms of ACKR1+/- ECs.
