# Supplementary material for: Single-cell transcriptome analysis reveals aberrant stromal cells and heterogeneous endothelial cells in alcohol-induced osteonecrosis of the femoral head
Source: Commun Biol. 2022 Apr 6;5:324. doi: 10.1038/s42003-022-03271-6 (PMC8987047; doi:10.1038/s42003-022-03271-6)
Supplement: Supplementary file 6 — Reporting Summary [file 42003_2022_3271_MOESM6_ESM.pdf]

nature portfolio

Corresponding author(s): Liang Zhao

Last updated by author(s): Feb 28, 2022

Reporting Summary

Nature Portfolio wishes to improve the reproducibility of the work that we publish. This form provides structure for consistency and transparency in reporting. For further information on Nature Portfolio policies, see our Editorial Policies and the Editorial Policy Checklist.

Statistics

For all statistical analyses, confirm that the following items are present in the figure legend, table legend, main text, or Methods section.

n/a	Confirmed	
☐	☒	The exact sample size (n) for each experimental group/condition, given as a discrete number and unit of measurement
☐	☒	A statement on whether measurements were taken from distinct samples or whether the same sample was measured repeatedly
☐	☒	The statistical test(s) used AND whether they are one- or two-sided<br>*Only common tests should be described solely by name; describe more complex techniques in the Methods section.*
☒	☐	A description of all covariates tested
☒	☐	A description of any assumptions or corrections, such as tests of normality and adjustment for multiple comparisons
☐	☒	A full description of the statistical parameters including central tendency (e.g. means) or other basic estimates (e.g. regression coefficient) AND variation (e.g. standard deviation) or associated estimates of uncertainty (e.g. confidence intervals)
☒	☐	For null hypothesis testing, the test statistic (e.g. F, t, r) with confidence intervals, effect sizes, degrees of freedom and P value noted<br>*Give P values as exact values whenever suitable.*
☒	☐	For Bayesian analysis, information on the choice of priors and Markov chain Monte Carlo settings
☒	☐	For hierarchical and complex designs, identification of the appropriate level for tests and full reporting of outcomes
☐	☒	Estimates of effect sizes (e.g. Cohen's d, Pearson's r), indicating how they were calculated

Our web collection on statistics for biologists contains articles on many of the points above.

Software and code

Policy information about availability of computer code

Data collection	This type of code was not used in our study
Data analysis	The specific information of the single-cell data analysis software involved in this study is shown in supplementary Table S4. The code used for data analysis in this study is referenced to the official tutorials of these software

For manuscripts utilizing custom algorithms or software that are central to the research but not yet described in published literature, software must be made available to editors and reviewers. We strongly encourage code deposition in a community repository (e.g. GitHub). See the Nature Portfolio guidelines for submitting code & software for further information.

Data

Policy information about availability of data

All manuscripts must include a data availability statement. This statement should provide the following information, where applicable:

- Accession codes, unique identifiers, or web links for publicly available datasets
- A description of any restrictions on data availability
- For clinical datasets or third party data, please ensure that the statement adheres to our policy

All data including reagent and software information associated with this study are in the paper or the supplementary materials. The raw data of scRNA-seq were posted to the SRA database (SRP361778). The processed sequencing data and the source data for graphs were posted to the Figshare (DOI: 10.6084/m9.figshare.19243722).

Field-specific reporting

Please select the one below that is the best fit for your research. If you are not sure, read the appropriate sections before making your selection.

☒ Life sciences ☐ Behavioural & social sciences ☐ Ecological, evolutionary & environmental sciences

For a reference copy of the document with all sections, see nature.com/documents/nr-reporting-summary-flat.pdf

Life sciences study design

All studies must disclose on these points even when the disclosure is negative.

Sample size	No sample-size calculation was performed. In this study, 7000-10,000 high-quality cells were detected in each sample, and a total of 11 samples were detect ed. These data can demonstrate the transcriptome charactertics and heterogeneity of local cells derived from the femoral head specimens
Data exclusions	In sc-RNA-seq analysis, cell s with a unique molecular identifier (UMI) number of less than 500, cells expressing fewer than 250 genes, cells with a mitochondrial gene expression ratio great er than 25% and cells with a gene/ UM I ratio less than 0.8 were filtered out.
Replication	All annotated cell clusters in ONFH, HOA and FNF groups appeared in all samples in each group
Randomization	N/A
Blinding	N/A

Reporting for specific materials, systems and methods

We require information from authors about some types of materials, experimental systems and methods used in many studies. Here, indicate whether each material, system or method listed is relevant to your study. If you are not sure if a list item applies to your research, read the appropriate section before selecting a response.

Materials & experimental systems

n/a	Involved in the study	
☐	☒	Antibodies
☐	☒	Eukaryotic cell lines
☒	☐	Palaeontology and archaeology
☐	☒	Animals and other organisms
☒	☐	Human research participants
☒	☐	Clinical data
☒	☐	Dual use research of concern

Methods

n/a	Involved in the study	
☒	☐	ChIP-seq
☐	☒	Flow cytometry
☒	☐	MRI-based neuroimaging

Antibodies

Antibodies used	Information on all antibodies used in this study including clone, species, manufacturer and catalog number is provided in supplementary Table S6
Validation	Validation of antibodies can be found on the manufacturer 's website

Eukaryotic cell lines

Policy information about cell lines

Cell line source(s)	L929(CL-0137) and THP-1(CL0233) were purchased from Procell (WuHan, China)
Authentication	All cell line were obtained from a company with the information of authentication(authenticated by STR)
Mycoplasma contamination	Cell line were not tested for Mycoplasma contamination
Commonly misidentified lines (See ICLAC register)	N/A

Animals and other organisms

Policy information about studies involving animals; ARRIVE guidelines recommended for reporting animal research

Laboratory animals	Balb/c mice, male, 8 weeks old.
Wild animals	Wild animals were involved in this study
Field-collected samples	Field-collected samples were involved in this study
Ethics oversight	This study was approved by the Nanfang Hospital Animal Ethic Committee (ref. NFYY- 2020-47)

Note that full information on the approval of the study protocol must also be provided in the manuscript.

Flow Cytometry

Plots

Confirm that:

☒ The axis labels state the marker and fluorochrome used (e.g. CD4-FITC).

☒ The axis scales are clearly visible. Include numbers along axes only for bottom left plot of group (a 'group' is an analysis of identical markers).

☒ All plots are contour plots with outliers or pseudocolor plots.

☒ A numerical value for number of cells or percentage (with statistics) is provided.

Methodology

Sample preparation	Amplified endothelial cells derived from femoral head specimens were used for flow cytometry sorting. Methods for cell extraction and amplification can be found in the materials and methods section
Instrument	Flow cytometry was performed on the MoFlo XDP (Beckman Coulter) platform
Software	Flow cytometry data were collected by the Summit software. The plot was done by FlowJO 10.0
Cell population abundance	Approximately 80% of all cells were identified as living cells, of which ACKR1+ ECs accounted for 4-10%
Gating strategy	Debris, dead cells and doublets cells were excluded by common gating strategy. ACKR1+ gates were set by comparing the negative control group

☒ Tick this box to confirm that a figure exemplifying the gating strategy is provided in the Supplementary Information.
